# The Perception of Contraceptive Practice Among Female Patients Treated With Isotretinoin in Saudi Arabia

**DOI:** 10.7759/cureus.69390

**Published:** 2024-09-14

**Authors:** Fatimah H Almarri, Mahdi Al Dhafiri, Reem A Albejais, Mohammed A Albaqshi, Waad D Alotaibi

**Affiliations:** 1 Medicine, King Faisal University, Al-Ahsa, SAU; 2 Dermatology, King Faisal University, Al-Ahsa, SAU; 3 Medicine, King Faisal University, Alhofuf, SAU; 4 Medicine, King Faisal University, Alahsa, SAU; 5 Medicine, King Faisal University, Hufof, SAU

**Keywords:** contraception, female, isotretinoin, perception, saudi arabia, teratogenicity

## Abstract

Background

Practicing and following a Pregnancy Prevention Program (PPP) is crucial to prevent isotretinoin-induced teratogenicity. Our study aims to assess the current practice of dermatologists about PPP when prescribing oral isotretinoin and the compliance of the patients toward contraceptive methods.

Methods

The research used a cross-sectional design and utilized a probability sampling method in Saudi Arabia. An online self-administered questionnaire was used to collect data that included female participants who were previously treated/being treated with isotretinoin for acne. Data was analyzed using SPSS (IBM Inc., Armonk, New York).

Results

Our study examined 359 female patients receiving isotretinoin treatment. Isotretinoin was primarily used for severe nodular forms of acne resistant to regular treatment 229 (63.8%). Regarding awareness, 326 (90.8%) were familiar with isotretinoin's teratogenic effects, and 112 (31.2%) were familiar with the Saudi Food and Drug Authority's Pregnancy Prevention Program. Physicians were among the most prevalent information sources 254 (71%). In terms of isotretinoin usage, 227 (63.2%) had one course. Regarding contraceptive practice, 167 (46.5%) were informed about the necessity of a negative pregnancy test before starting treatment, and 29 (50.9%) of those who received more than two courses had good awareness.

Conclusion

The study identified a notable awareness gap among the participants, as over two-thirds demonstrated a poor awareness level regarding isotretinoin. A significant proportion of participants were informed about the importance of contraceptive practices, including the necessity of a negative pregnancy test before, during, and after treatment.

## Introduction

Acne vulgaris is an inflammatory disorder of the sebaceous glands, ducts, and hair follicles that may last for years [1,2]. It frequently affects the face, back, and chest and has a significant familial tendency. Oral Isotretinoin is a vitamin A-based acne treatment used for moderate-severe acne and severe cystic acne [3]. The most frequent side effects of isotretinoin are temporary exacerbation of acne, dry and fragile skin, and increased risk of photosensitivity and sunburn [4]. Dyslipidemia, altered liver function, epistaxis, joint and back discomfort are further potential adverse effects [5]. The incidence of congenital abnormalities in fetuses exposed to medicines during intrauterine life ranges from 25% to 35% [6]. According to the research findings, there is evidence suggesting a correlation between the administration of isotretinoin and a diverse array of congenital anomalies [7]. These anomalies encompass various malformations affecting the neurological and cardiovascular systems, as well as craniofacial structures [8]. Additionally, there is a potential association with adverse outcomes, including psychomotor and cognitive impairments, intellectual disabilities, and premature birth [9]. Like in many countries, a Pregnancy Prevention Program (PPP) was put into place by the Saudi Food and Drug Authority (SFDA) to protect female patients from the teratogenic effects of their fetuses in case they become pregnant during the treatment with isotretinoin [4]. The above program consists of six parts: an isotretinoin patient information sheet, a contraception education sheet, a delivery guide for pharmacists, a prescription guide and checklist for physicians, and a permission consent form for women of reproductive age. A negative pregnancy test and a proper contraceptive method start before oral isotretinoin and during the course and for one month after stopping the treatment [10]. Our aim in this study is to assess the current practice of dermatologists about PPP when prescribing oral isotretinoin and the compliance of the patients toward contraceptive methods.

## Materials and methods

The study was designed as a cross-sectional study and was conducted using a probability sampling method in the Kingdom of Saudi Arabia. The study took six months to complete. An online self-administered questionnaire using Google Forms (Google, Mountain View, California) was distributed among the female population residing in Saudi Arabia who had previously been treated or were currently being treated with isotretinoin for acne. The questionnaire addressed specific objectives, including whether participants had received an explanation about the relationship between teratogenicity and isotretinoin use before starting treatment, whether they practiced proper contraceptive measures with good compliance, and whether they had undergone a pregnancy test before and during treatment with a confirmatory negative result. The target population for the study comprised Saudi females treated or being treated with isotretinoin for acne. 

Data analysis involved the extraction, revision, and coding of data, followed by input into the statistical program SPSS version 22 (IBM Inc., Armonk, New York). Two-tailed tests were used, and statistical significance was considered when the p-value was less than 0.05. Awareness and perception were assessed using aggregate scores, with patients scoring less than 60% of the maximum score classified as having a low degree of awareness. Those with an overall score of 60% or more were considered to have a good overall awareness level. Descriptive analyses, including frequency and percent distribution, were conducted for all variables, including patients' bio-demographic information and level of education. Additionally, female patients' awareness regarding isotretinoin, their frequency and duration of treatment, and contraceptive practices were tabulated, and cross-tabulations were performed using exact probability tests and the Person's Chi-squared test to evaluate characteristics related to parents' knowledge of and perception of isotretinoin.

Inclusion and exclusion criteria 

Saudi females living in Al-Ahsa who treated or being treated with isotretinoin for acne were included in the study. Male gender, non-Saudi patients, any female not treated with isotretinoin, participants receiving isotretinoin for health conditions other than acne, and participants not living in Al-Ahsa were excluded from the study. 

## Results

The study had a cohort of 359 female individuals who received or are currently receiving treatment with isotretinoin. The age of the patients varied from 16 to 60 years, with a mean age of 22.1 ± 13.9 years. A total of 253 (70.5%) were university graduates, and 84 (23.4%) had a secondary level of education. As for marital status, 261 (72.7%) were single patients and 85 (23.7%) were married (Table 1).

**Table 1:** Personal characteristics of study female patients treated with isotretinoin

Personal data	No	%
Age in years		
16-25	241	67.1%
26-35	87	24.2%
36-45	25	7.0%
46+	6	1.7%
Education level		
Below secondary	7	1.9%
Secondary	84	23.4%
University	253	70.5%
Post-graduate	15	4.2%
Marital status		
Single	261	72.7%
Married	85	23.7%
Divorced / widow	13	3.6%

Regarding indications of isotretinoin, 229 (63.8%) reported it was used for refractory severe nodular forms of acne, and 83 (23.1%) said it was used for acne with oily skin with or without dilated pores, while 37 (10.3%) told it was used for all types of acne. With regard to the maximum daily dose, 125 (34.8%) reported that it is 30-40 mg/day, 90 (25.1%) reported 10-20 mg/day, and 102 (28.4%) don't remember. Exactly 326 (90.8%) knew that isotretinoin has a teratogenic effect, and 112 (31.2%) had heard of SFDA's Pregnancy Prevention Program that established guidelines for the Isotretinoin use among females of conceiving age group (15-49 years; Table 2).

**Table 2:** Female patients' perception and awareness of isotretinoin

Perception and awareness	No	%
Indication for isotretinoin		
Severe nodular forms of acne resistant to regular treatment	229	63.8%
Acne with oily skin with or without pores	83	23.1%
Acne on the cheek	10	2.8%
All forms of Acne	37	10.3%
The daily maximum dose of isotretinoin		
10 to 20 mg	90	25.1%
30 to 40 mg	125	34.8%
50 to 60 mg	39	10.9%
More than 70 mg	3	.8%
Don't know	102	28.4%
The teratogenic effect is SE		
Yes	326	90.8%
No	12	3.3%
Don't know	21	5.8%
Have you ever heard of the Saudi Food and Drug Authority's (SFDA) Pregnancy Prevention Program (PPP) that established guidelines for isotretinoin use among females of reproductive age group (15-49 years)?		
Yes	112	31.2%
No	247	68.8%

**Figure 1:**
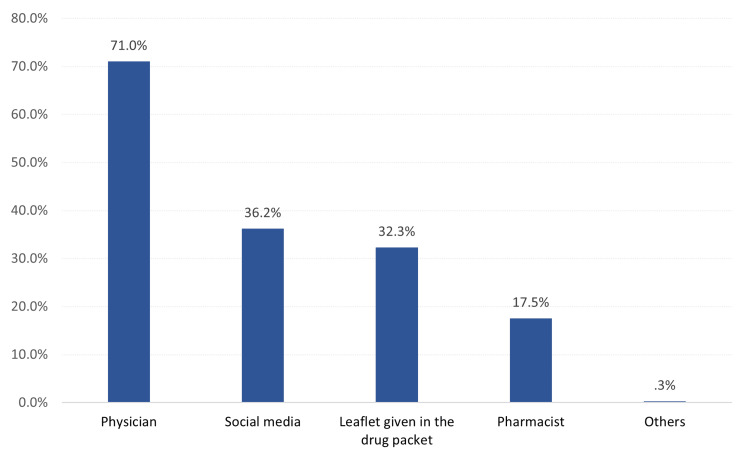
Source of information about isotretinoin among female patients

Source of information (teratogenic) about isotretinoin among female patients. The most reported sources of information were physicians (71%), followed by social media (36.2%), drug leaflets (32.3%), and pharmacists (17.5%; Figure 1). 

Regarding the number of the received courses of isotretinoin treatment, q227 (63.2%) of the participants had one course of isotretinoin, 75 (20.9%) had two courses, and 57 (15.9%) had more than two courses. As for the duration of isotretinoin intake, 150 (41.8%) had the treatment for 5-7 months, 127 (32.4%) had it for two to four months, and 52 (14.5%) had it for 8-10 months, but 30 (8.4%) had isotretinoin for 11-12 months. A total of 342 (95.3%) received isotretinoin with a doctor’s prescription, while 17 (4.7%) were taking isotretinoin without prescription (Table 3). 

**Table 3:** Female patients' frequency and duration of isotretinoin

Treatment frequency	No	%
Number of treatment courses with isotretinoin		
1 course	227	63.2%
2 courses	75	20.9%
>2 courses	57	15.9%
Duration of taking isotretinoin (months)		
2-4 months	127	35.4%
5-7 months	150	41.8%
8-10 months	52	14.5%
11-12 months	30	8.4%
Are you taking isotretinoin with a doctor's prescription?		
Yes	342	95.3%
No	17	4.7%

Exactly 63.2% of the study female patients reported that they signed the Exactly 63.2% of the study female patients reported that they signed the patient’s consent form before beginning the treatment. As for contraceptive practice and physician role in patients’ education, 46.5% of the study female patients on isotretinoin were informed about the necessity a negative pregnancy test before starting treatment, 41.8% were informed about the necessary of a negative pregnancy test during treatment, 37% were informed about the necessary of negative pregnancy test that is necessary 5 weeks after stopping the treatment, 35.4% of participants received information regarding the significance of employing two contraceptive methods prior to initiating treatment. Similarly, 34.8% were informed about the importance of using two contraceptive methods four weeks after discontinuing the treatment. Additionally, 33.4% of participants were provided with information regarding the importance of utilizing two contraceptive methods during the course of treatment (Table 4).

**Table 4:** Contraceptive practice among female patients treated with isotretinoin

Contraceptive practice items	Yes		No	
	No	%	No	%
Have you been informed about the necessity of a negative pregnancy test before starting treatment?	167	46.5%	192	53.5%
Have you been informed about the necessity of negative pregnancy tests during the treatment?	150	41.8%	209	58.2%
Have you been informed that a negative pregnancy test is necessary 5 weeks after stopping the treatment?	133	37.0%	226	63.0%
Have you been informed about the significance of using dual contraception techniques before starting treatment?	127	35.4%	232	64.6%
Have you been informed about the significance of using dual contraception techniques during the treatment?	120	33.4%	239	66.6%
Have you been informed about the significance of using dual contraception techniques 4 weeks after stopping the treatment?	125	34.8%	234	65.2%
Have you signed the patient's consent form before beginning the treatment?	227	63.2%	132	36.8%

A total of 239 (66.6%) had an overall poor general awareness regarding isotretinoin, while 120 (33.4%) had a good awareness level. Exactly 50.9% of patients who received more than two courses of the treatment had an overall good awareness level compared to 29.5% of others who received only one course with record statistical significance (p=o.009). Also, good awareness was reported higher among female patients who were informed about contraceptive use and pregnancy testing at different phases of the treatment (Table 5).

**Table 5:** Factors associated with female patients' awareness of isotretinoin treatment P: Pearson X2 test; $: exact probability test; * p<0.05 (significant)

Factors		Awareness level				p-value
		Poor (239)		Good (120)		
		No	%	No	%	
Age in years	16-25	167	69.3%	74	30.7%	.076^$^
	26-35	52	59.8%	35	40.2%	
	36-45	14	56.0%	11	44.0%	
	46+	6	100.0%	0	0.0%	
Educational level	Below secondary	6	85.7%	1	14.3%	.716^$^
	Secondary	54	64.3%	30	35.7%	
	University	169	66.8%	84	33.2%	
	Post-graduate	10	66.7%	5	33.3%	
Marital status	Single	177	67.8%	84	32.2%	.637
	Married	53	62.4%	32	37.6%	
	Divorced / widow	9	69.2%	4	30.8%	
Number of treatment courses with isotretinoin	1 course	160	70.5%	67	29.5%	.009*
	2 courses	51	68.0%	24	32.0%	
	> 2 courses	28	49.1%	29	50.9%	
Duration of taking Isotretinoin (months)	2-4 months	84	66.1%	43	33.9%	.826
	5-7 months	102	68.0%	48	32.0%	
	8-10 months	32	61.5%	20	38.5%	
	11-12 months	21	70.0%	9	30.0%	
Have you been informed about the necessity of a negative pregnancy test before starting treatment?	Yes	94	56.3%	73	43.7%	.001*
	No	145	75.5%	47	24.5%	
Have you been informed about the necessity of negative pregnancy tests during the treatment?	Yes	88	58.7%	62	41.3%	.007*
	No	151	72.2%	58	27.8%	
Have you been informed that a negative pregnancy test is necessary 5 weeks after stopping the treatment?	Yes	72	54.1%	61	45.9%	.001*
	No	167	73.9%	59	26.1%	
Have you been informed about the significance of using dual contraception techniques before starting treatment?	Yes	65	51.2%	62	48.8%	.001*
	No	174	75.0%	58	25.0%	
Have you been informed about the significance of using dual contraception techniques during the treatment?	Yes	60	50.0%	60	50.0%	.001*
	No	179	74.9%	60	25.1%	
Have you been informed about the significance of using dual contraception techniques 4 weeks after stopping the treatment?	Yes	65	52.0%	60	48.0%	.001*
	No	174	74.4%	60	25.6%	
Have you signed the patient's consent form before beginning the treatment?	Yes	140	61.7%	87	38.3%	.010*
	No	99	75.0%	33	25.0%	

## Discussion

This study provides a comprehensive insight into the complex landscape of dermatologists' practices and patients' adherence to the Pregnancy Prevention Program. The presence of educated, single patients underscores the importance of tailored contraceptive counseling, as many of these women may be of childbearing age and sexually active [11].

Additionally, the study reveals that healthcare professionals are the primary source of information about isotretinoin teratogenic effect and the importance of following a PPP, consistent with the study conducted in Jordan by Jarab et al. (2022) [12]. Prior research delves into the perspectives of healthcare providers, regarding contraceptive counseling for isotretinoin users in Saudi Arabia, offering insights into their role in patient education [13]. Thus, the study's implications extend to the need for enhanced training and awareness among dermatologists and other healthcare providers regarding the importance of thorough patient education when prescribing isotretinoin [14].

Approximately 63.2% of the participants in the study reported signing the patient's consent form before starting treatment, indicating a relatively high risk of teratogenicity associated with isotretinoin. However, there are notable gaps in patient education related to contraceptive practices and the role of physicians. A substantial proportion of patients were not adequately informed about the necessity of negative pregnancy tests before starting treatment, during treatment, and after stopping treatment. Similarly, a significant percentage of patients were not informed about the importance of using two contraceptive methods before treatment initiation, during treatment, and after treatment completion. These findings underscore the need for dermatologists and healthcare providers to enhance their commitment to educate patients about the PPP and contraceptive methods [15]. The study by Al-Harbi et al. (2010) presents the acne awareness and practices of female isotretinoin users in Saudi Arabia, shedding light on potential cultural and geographical variations [16].

The study by Alshaalan et al. (2022) showed the impact of a standardized patient education program on isotretinoin-related teratogenic risk awareness among female patients, providing insights into educational interventions [17].

Regarding the factors influencing the level of awareness among participants, age and educational level show no significant role. Interestingly, the number of treatment courses with isotretinoin emerged as a significant factor affecting awareness, highlighting the importance of continuous patient education, especially for those undergoing multiple courses of treatment. The study conducted by Ibrahim et al. (2021) investigates how awareness of pregnancy prevention measures among patients undergoing multiple isotretinoin courses evolves over time, addressing the importance of continuous education [4].

Encouragingly, patients who were informed about the necessity of negative pregnancy tests at different stages of treatment demonstrated significantly better awareness, emphasizing the significance of clear communication between healthcare providers and patients. The study conducted by Thielitz et al. (2013) examined the variability in dermatologist-patient communication regarding isotretinoin's teratogenic risk, shedding light on regional differences [18].

These results suggest a need for standardized and consistent patient education practices among dermatologists prescribing isotretinoin. Healthcare providers should ensure that patients receive comprehensive information about contraceptive methods and the importance of regular pregnancy tests. This study highlights an opportunity to enhance patient awareness and potentially reduce the risk of teratogenic effects associated with isotretinoin use [19]. Future efforts in healthcare practice and policy should focus on improving patient education and compliance with contraceptive methods in the context of isotretinoin treatment [20], ultimately promoting safer and more effective patient care in Saudi Arabia. Despite its valuable insights, this study has some limitations worth noting. Firstly, the research focused exclusively on female patients in Saudi Arabia, which could restrict the results' applicability to different cultural and geographic settings. Second, the study used self-reported information, which might be impacted by biases such social desirability and memory distortion. Additionally, the research primarily examined patient awareness and practices; thus, it did not delve into the perspectives of healthcare providers or explore potential barriers to effective patient education.

## Conclusions

The research underscores the essential role of healthcare professionals, particularly dermatologists, in educating patients about the teratogenic risks associated with isotretinoin and the importance of effective contraceptive practices during treatment. The demographic diversity of the patient population highlights the need for tailored patient education strategies. Future studies should explore the perspectives of healthcare providers, investigate potential barriers to patient education, and seek to establish causal relationships between various factors and patient awareness levels. 

## Appendices

**Table 6:** Appendices

	Age (Specify your age in years):
▪ No formal education ▪ Up to high school ▪ Bachelor's degree ▪ Masters and above	Education:
▪ Single/Unmarried ▪ Married ▪ Divorced/Widowed	Marital status:

Which of the following is indication for isotretinoin (Roaccutane/Xeractan/Curacne)?	▪ All forms of acne ▪ Acne with oily skin with or without pores ▪ Acne on the cheek ▪ Severe nodular forms of acne resistant to regular treatment
What is the daily maximum dose of Isotretinoin?	▪ 10 to 20 mg ▪ 30 to 40 mg ▪ 50 to 60 mg ▪ More than 70 mg
One of the side effects of the Acne? (Kindly answeryes/no/Do not know)	Teratogenic effect: Yes No
(It affects fetus if you take during pregnancy time) Whatis your major source of information regarding side effectsof Isotretinoin?	Doctors ▪ Pharmacists ▪ Internet/social media ▪ Leaflet given in the drug packet
Have you ever heard of Saudi Food and Drug Authority (SFDA) — Pregnancy Prevention Program (PPP) thatestablished guidelines for the Isotretinoin use amongfemales of reproductive age group (15 — 49 years)?	Yes ▪ No
o Are you currently on Isotretinoin or used Isotretinoin in the past one year (Roaccutane/Xeractan/Curacne)?	Yes ▪ No
Number of treatment courses with isotretinoin	One Two More than two
Are you taking isotretinoin with doctor's prescription?	▪ Yes ▪ NO
What is the duration of treatment? (Specify in months)	
Have you been informed about the necessary of negative pregnancy test before to start treatment?	▪ Yes ▪ No ▪ Not sure
Have you been informed about the necessary of negative pregnancy test during the treatment?	▪ Yes ▪ No ▪ Not sure
Have you been informed about the necessary of negative pregnancy test that is necessary 5 weeks after stoppingthe treatment?	▪ Yes ▪ No ▪ Not sure
Have you been informed about importance of use of two contraceptive methods before to start treatment?	▪ Yes ▪ No ▪ Not sure
Have you been informed about importance of use of two contraceptive methods during the treatment?	▪ Yes ▪ No ▪ Not sure
Have you been informed about importance of use of two contraceptive methods 4 weeks after stopping the treatment?	▪ Yes ▪ No ▪ Not sure
Have you signed the patient's consent form before beginning the treatment?	▪ Yes ▪ No ▪ Not sure
